# Spectroscopic and biochemical correlations during the course of human lens aging

**DOI:** 10.1186/1471-2415-6-10

**Published:** 2006-03-06

**Authors:** Mala Ranjan, Sashidhar Rao Beedu

**Affiliations:** 1 Department of Biochemistry, University College of Science, Osmania University, Hyderabad- 500 007 (A.P.), India

## Abstract

**Background:**

With age, the human lens accumulates variety of substances that absorbs and fluorescence, which explains the color of yellow, *brunescent *and *nigrescent *cataract in terms of aging. The aim of this study was to assess lens fluorophores with properties comparable to those of advanced glycated end products (AGEs) in relation to age in human lenses. These fluorescent compounds are believed to be involved in the development of cataract.

**Methods:**

Spectroscopic (UV-Vis-NIR) and fluorescence photography (CCD-Digital based image analysis) studies were carried out in randomly selected intact human lenses (2–85 years). AGE-like fluorophores were also measured in water soluble and insoluble (alkali soluble) fractions of human lenses (20–80 years).

**Results:**

Our experimental findings suggest that there was a progressive shift in the absorbance characteristic of intact lens in the range of *λ_210 nm_*-*λ_470 nm_*. A relative increase in the absorptivity at *λ_(511–520 nm)_*, with age, was also observed. In addition, the ratio of absorptivity at λ_(511–520 nm)_* versus *the maximum absorbance recorded at blue-end cut-off (210–470 nm) was also found to increase, with age. The fluorescent intensity in the intact lens at both UV-B (*λ*_Ex312 nm_) and UV-A *(λ_Ex365 nm_*) were found to be positively correlated (*r*^2 ^= 0.91 & 0.94, respectively; Confidence interval 95%) upto 50 years of age. In addition, a concomitant changes in AGE- like fluorophores were also observed in the processed lens samples (soluble and insoluble fractions) along the age. A significant increase in the concentration of AGE- like fluorophores, both in intact and processed lens was observed during the period of 40 – 50 years.

**Conclusion:**

Based on the present investigation, it was concluded that significant changes do occur in the AGE-like fluorophores of human lenses during the period of 40–50 years.

## Background

The young human lens is colorless and transmits almost 100% of the incident light. With age, photochemical mediated interactions results in the development of coloration of lenses and formation of fluorescent biochemical products [1]. Light mediated damage to the lens proteins can only occur due to the presence of light absorbing chromospheres. It has been reported earlier that the young lenses shows higher absorbance at 280 nm due to the presence of aromatic amino acids like tryptophan and tyrosine constituent of crystallins [2]. During the process of aging, the human lenses become progressively more yellow and fluorescent [3-5]. The age-related increase in lens coloration and fluorescence is associated with the major proteins of the lens, the crystallins, which are particularly prominent in the lens nucleus [4,5]. The lens proteins are as old as the individual since there is little or no turn over of these proteins [6]. Numerous chemical and photochemical processes may account for these changes, which include the (i) photochemical modification of tryptophan [7] (ii) lipid peroxidation [8] and (iii) chemical linking of sugar or ascorbic acid through the Maillard reaction [9,10].

The Maillard reaction, a non-enzymatic reaction between ketones or aldehydes and primary amino groups of macromolecules, contributes to the aging of proteins and to complications associated with diabetes [11-13]. Advanced glycated end products (AGEs) were originally characterized by a yellow-brown fluorescent color and their ability to form cross-links with and between amino groups [14]. Fluorescence is one of the qualitative properties classically used to estimate AGE formation in addition to their brown coloration. There is considerable evidence that AGE of lens proteins are involved in browning and fluorophore formation in the lens [15,16]. Several different AGEs (fluorescent cross-links and non-fluorescent cross links) have been detected in the human lenses, mainly by immunochemical methods using both polyclonal and monoclonal antibodies [17-21] in normal, aged and cataractous lenses. The identified fluorescent AGEs species in lens include pentosidine[22], pyralline[24], crossaline[23], vesperlysine, and argpyrimidine[24]. Additionally, Franke *et al*., [17] have reported the presence of pentosidine and imidazolone, in cataractous lenses. Methylglyoxal-derrived hyroimidiazolone AGEs are the major glycation adducts found in lens proteins compare to argpyrimidine and pentosidine [25]. Undoubtedly, there are a number of AGE-like fluorophore that are formed in human lenses during the course of aging. The direct relationship between the amount of AGEs and increased yellowing of lens was reported earlier by Das *et al*., [26] using synchronous fluorescence (SF) method. The SF spectra of human lens sample closely resembled those of *in vitro *AGEs derived from incubation of BSA with glucose.

In addition, post-translational modifications by kynurenine (tryptophan-metabolite), 3-hydroxykynurenine glucoside (3-OHKG) with lens crystallins [27-29] as aging fluorophores, have been considered as biomarker for aging of lens. However, no spectroscopic studies have been carried out on the aging of human lenses, which particularly deals with the early onset of cataractogenesis in terms of AGE-like fluorophores formation, which can be correlated with biochemical and photo-biological events occurring during our lifetime.

The present experimental investigation was designed to assess AGE-like fluorophores with properties comparable to those of advanced glycated end products (AGEs) in relation to age in human lenses by spectroscopic approach. This study was undertaken to address the early onset of cataractogenesis in terms of AGE-like fluorophore formation in human lens.

## Methods

Human lenses (2–85 years) were obtained from Ramayamma International Eye Bank (Member of the International Federation of Eye Banks & Eye Bank Association of India)L V Prasad Eye Institute, Hyderabad, India. This study was undertaken with the approval of the ethics committee of L V Prasad Eye Institute, Hyderabad, India. The informed consent was also obtained for collecting those cadaver lenses through the Eye Bank of L V Prasad. They were stored at -80°C, until further use.

### Chemicals

Bovine serum albumin {(BSA) Essential fatty acid & globulin free}, Glucose (Dextrose; corn sugar), L-arginine were purchased from Sigma Chem. Co. St. Louis, USA. Crystalline D- ribose from Hi Media, India. Amino acids, L-tyrosine and L-histidine were from Eastman Kodak Company, Rochester, N.Y and L-lysine monohydrochloride from M/s Sarabhai M. Chemicals, Baroda, India. All other reagents used were of analytical grade.

### Spectral measurements

Human lenses (2–85 years, n = 45) with varying degree of yellow- brown coloration were randomly selected for the spectral analysis and grouped as shown in Table 1.

**Table 2:** Relative change in the ratio of absorbance observed in the region of 511–520 nm versus absorbance recorded at blue-end cut-off region.

Age of lens (year)	Absorbance^# ^in the region of λ511–520 nm	Ratio of Absorbance^# ^Absorbance at λ511–520 nm λ_max _at blue-end cut-off
2	0.25	0.25/1.5 = 0.16
15	0.6	0.6/3.12 = 0.192
20	1.0	1.0/3.5 = 0.28
30	1.2	1.2/3.8 = 0.315
45	1.3	1.3/4= 0.325
60	1.35	1.35/> 4*
65	2.6	2.6/> 4*
75	1.18	1.18/3.5 = 2.96
80	2.3	2.3/> 4*
85	2.75	2.75/> 4*

^# ^Mean value

* Values not calculated as absorbance was > 4.0

**Table 1:** 

**Age group (years)**	**Number lenses (n)**
2	2
15–17	5
20–23	5
30–35	5
45–47	5
57–60	5
65–70	3
75 – 85	15

Intact lenses were subjected to fiber optic based digital UV-Vis-NIR spectrophotometer (Ocean Optics, Netherlands) analysis. This system uses the continuous output of Xenon lamp (200–1000 nm) and is based on 1024 diode array detector system. This detector is capable of collecting full wavelength spectra with good signal to noise ratio at an integration on time of 1 milli sec. The excitation light is led into the sample chamber through a fiber optic bundle, and the transmitted light is then collected by a second fiber optic bundle positioned at an angle of 180° to the excitation source. The excitation and transmitted light are collimated by a set of focusing lens on the either side of the sample chamber. The spectra from the intact human lenses were taken against the dark (0% transmittance) and reference (100%T) spectra. The acquired digital data was analyzed using the software (OOI, Basic Acquisition Software, Version 1.52.) supplied along with the instrument.

### Fluorescence studies

Forty lenses (20–80 years) were selected for fluorescent studies. Fluorescent intensity (density) in the intact human lenses were measured peak volume at both UV-B and UV-A (*λ_Ex312 nm _& λ_Ex365 nm_*,) respectively with an emission in the range of 380 nm to 470 nm, using highly sensitive charged coupled device (CCD) based digital image analyzer (UVItec) Cambridge, U.K. Volume of the digital image due to fluorescent intensity (density) was analyzed by using UVI Image Acquisition and Analysis Software.

### Digital Image Analysis System

This system consists of a high-resolution CCD-based (charged coupled device) camera (monochrome type) with absorbance and fluorescence facility. The CCD-camera has an optical zoom lens (12.5 × 75 mm/f1.8) along with 49-mm+1 dioptres close-up lens. The sensitivity of the camera is 10^-5 ^lux, with negligible signal to noise ratio (< 30 db). The CCD camera is housed in a light-tight compact cabinet over a trans-illuminator. The camera is equipped with UV & IR interference filter. The acquired image is displayed on an in-built LCD screen (resolution – 8 bit, 256 gray level images). The image acquisition is base on real time integration in the range of 0.04 to 10 seconds. The image acquisition system is linked to Intel Pentium 4 processor base computer, loaded with UVi-tech image processing and store software (64-bit data software). The digitized image has a resolution of 752(H) × 582 (V) pixels. The trans-illuminator consists of dual wavelength (312 and 365 nm) UV lamps with out put light intensities of 8 mW/cm^2^.

The digital image acquisition was achieved by placing the intact lens on flat surface of the trans-illuminator, housed in the light-tight cabinet. For florescence studied, the sample was excited individually from the base at both UV-B & UV-A (*λ_Ex312 nm _& λ_Ex365 nm_*) region of the electromagnetic spectrum. Lens image formed due to the emission (range 380 nm to 470 nm) was acquired by real time integration at 0.2 seconds for 312 nm and 0.04 seconds for 365 nm. Annotated images were saved in PC compatible file format (tiff file) in a floppy. Later, the digital images of the intact lens were analyzed by the software for determining the net fluorescence intensity (density), which was measured as peak volume.

### Processing of human lens samples

Lenses (20–80 years) were homogenized individually in 20 mM Phosphate buffer pH 7.4 (10% w/v), centrifuged at 10,000 × g for 30 min at 4°C, and separated into supernatant and precipitate. The supernatant referred to as the "water soluble" fraction and precipitate as the "water insoluble" fraction. An aliquot of the insoluble protein (1–3 mg) was solublized in 200–500 μL of 0.1 N NaOH. Both fractions were used for fluorescent studies for the estimation of AGE-like fluorophores. Protein was estimated by the method of Lowry *et al*., [30] using BSA as reference standard. For fluorescent studied 1 mg/mL of protein was taken.

### Chemical synthesis of AGE-like fluorophores

Synthetic AGE-like fluorophores were prepared for comparison with lens AGE-like fluorophores [31].

(i) BSA (1 mg/mL of 0.1 N NaOH) was used as control blank for fluorescent studies. (ii) BSA-AGE was prepared by modified method of Nakagawat, *et al*., [31]. It was prepared by incubating 1.5 μ mole BSA with 1.6 mmole glucose, 0.7 mmole ribose in 5 mL of 0.4 M phosphate buffer, pH 7.4. The samples were processed under sterile condition using the Laminar flow hood. The vials were sealed and placed in an air-circulating incubator, at 37°C for 10 weeks. After incubation, mixture was dialyzed and concentrated using spin column (Ultrafree-MC filters, molecular weight cut-off limit – 10 kDa, Sigma Chem. Co. St. Louis, USA.). The degree of glycation was checked by trinitobenzene sulfonic acid method [32]:

% Conjugation=[(Conc. of ε−amino group in BSA)−(Conc. of ε −amino group in BSA after glycation)]Conc. of ε −amino group in BSA
 MathType@MTEF@5@5@+=feaafiart1ev1aaatCvAUfKttLearuWrP9MDH5MBPbIqV92AaeXatLxBI9gBaebbnrfifHhDYfgasaacH8akY=wiFfYdH8Gipec8Eeeu0xXdbba9frFj0=OqFfea0dXdd9vqai=hGuQ8kuc9pgc9s8qqaq=dirpe0xb9q8qiLsFr0=vr0=vr0dc8meaabaqaciaacaGaaeqabaqabeGadaaakeaacqGGLaqjcaaMc8ocbaGamqhG=neadjad0b4FVbWBcWaDa+NBa4MamqhG=PgaQjad0b4F1bqDcWaDa+3zaCMamqhG=fgaHjad0b4F0baDcWaDa+xAaKMamqhG=9gaVjad0b4FUbGBcqGH9aqpdaWcaaqaamaadmaabaWaaeWaaeaacqWFdbWqcqWFVbWBcqWFUbGBcqWFJbWycqGGUaGlcaaMc8Uae83Ba8Mae8NzayMaaGPaVlabew7aLjabgkHiTiab=fgaHjab=1gaTjab=LgaPjab=5gaUjab=9gaVjaaykW7cqWFNbWzcqWFYbGCcqWFVbWBcqWF1bqDcqWFWbaCcaaMc8Uae8xAaKMae8NBa4MaaGPaVlab=jeacjab=nfatjab=feabbGaayjkaiaawMcaaiabgkHiTmaabmaabaGae83qamKae83Ba8Mae8NBa4Mae83yamMaeiOla4IaaGPaVlab=9gaVjab=zgaMjaaykW7cqaH1oqzcaaMc8UaeyOeI0Iae8xyaeMae8xBa0Mae8xAaKMae8NBa4Mae83Ba8MaaGPaVlab=DgaNjab=jhaYjab=9gaVjab=vha1jab=bhaWjaaykW7cqWFPbqAcqWFUbGBcaaMc8Uae8NqaiKae83uamLae8xqaeKaaGPaVlab=fgaHjab=zgaMjab=rha0jab=vgaLjab=jhaYjaaykW7cqWFNbWzcqWFSbaBcqWF5bqEcqWFJbWycqWFHbqycqWF0baDcqWFPbqAcqWFVbWBcqWFUbGBaiaawIcacaGLPaaaaiaawUfacaGLDbaaaeaacqWFdbWqcqWFVbWBcqWFUbGBcqWFJbWycqGGUaGlcaaMc8Uae83Ba8Mae8NzayMaaGPaVlabew7aLjaaykW7cqGHsislcqWFHbqycqWFTbqBcqWFPbqAcqWFUbGBcqWFVbWBcaaMc8Uae83zaCMae8NCaiNae83Ba8Mae8xDauNae8hCaaNaaGPaVlab=LgaPjab=5gaUjaaykW7cqWFcbGqcqWFtbWucqWFbbqqaaaaaa@DCBC@

(iii) Chemical synthesis of amino acids mixture -AGE: Here we have selected few protein amino acids, which are present in the sequence of human γ-crystallins. These amino acids were selected because of their high susceptibility to glycation/AGE formation in the course of cataract development. To mimic the fluorescent AGEs like crosslinks (pentosidine, argpyrimidine, pentosidine, pyropyridine etc.), we incubated following amino acids mixture: 9.5 μ mole of lysine, 100 μ mole of arginine, 23 μ mole histidine and 67 μ mole of tyrosine along with 1.6 mmole glucose, 0.7 mmole ribose in 5 mL of 0.4 M phosphate buffer, pH 7.4 under the same experimental conditions as mentioned above. A "zero" day sample of above mentioned amino acid mixture with sugars was used as control blank.

### Fluorescence measurements

Fluorescent measurements were performed in total as well as both fractions of human lens samples, using a spectrofluorimeter (Perkin-Elmer, LS-3B, Norwalk, NJ, USA). AGE-like fluorophores were measured as described earlier [33] in sample, experimentally synthesized AGE-like fluorophores as well as in control blank (BSA /1 mg/mL in 0.1 N NaOH). These AGE-like fluorophores were measured at their respective excitation and emission wavelength, in the following order:

AGE (λ_Ex347 nm_/λ_Em415 nm_); pentosidine (λ_Ex335 nm_/λ_Em385 nm_); pentodilysine (λ_Ex366 nm_/λ_Em440 nm_); crossline (λ_Ex379 nm _/λ_Em463 nm_); pyropridine(λ_Ex370 nm _/λ_Em455 nm_); argpyrimidine(λ_Ex320 nm_/λ_Em382 nm_). Results are expressed as fluorescence intensity/mg of lens protein.

### Statistical analysis

The data was statistically analyzed by using Sigma-plot software version 5.0. The test of significance was based on Student's *t*- test.

## Results

**Figure 1:**
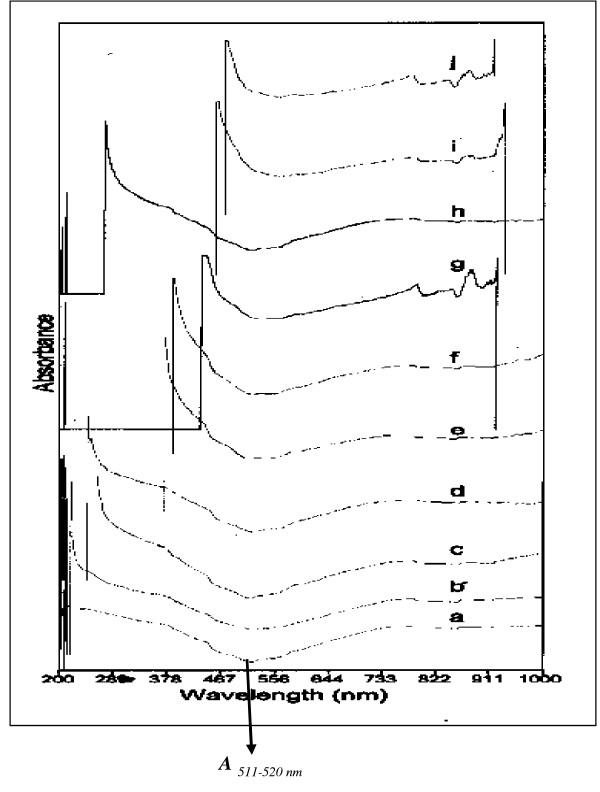
A typical superimposed representative absorption spectrum of intact human lenses (2–85 years). a- 02, b- 15, c- 20, d- 30, e- 45, f- 60, g- 65 (diabetic), h- 75, i- 80, j-85 years old lenses. A progressive shift in the wavelength of absorption from λ_210 nm _to λ_470 nm _was observed. An increase in absorbance was recorded (range 0.25 to = 3.0 - not shown on *y - axis*) at λ_511–520 nm _with age.

The absorption spectra of intact human lenses from various age groups (2–85 years) are given in Figure 1. There was a progressive shift of absorbance in the region of λ_210_-λ_470 nm_, in relation to age (exception 75 year old lens). Table 2 clearly indicates a relative increase in the absorptivity at λ_(511–520 nm_), with age. The ratio of absorptivity at λ_(511–520 nm_*) versus *the maximum absorbance recorded at blue-end cut-off (210–470 nm) was also found to increase, with age.

**Figure 2:**
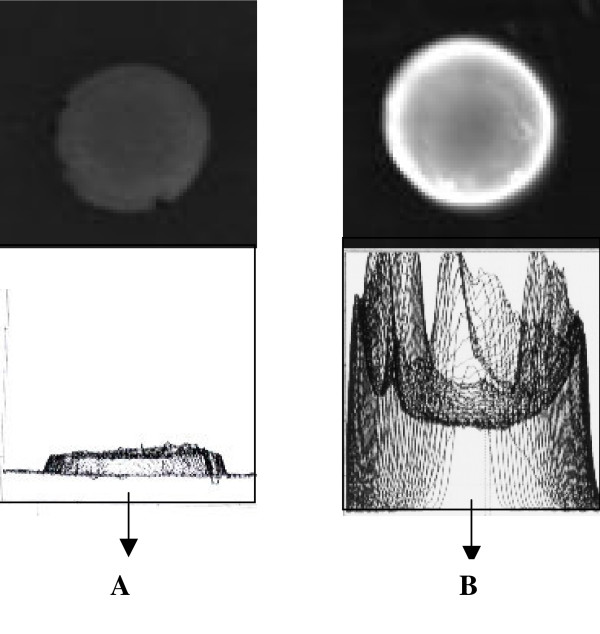
Digital based photographic analysis of a typical intact human lens (**A**)- 20 year (control) and (**B**) 60 year cataractous, at λ_Ex312 nm _*/λ *_Em380–470nm. _The fluorescence intensity (density) increased with age.

**Figure 3:**
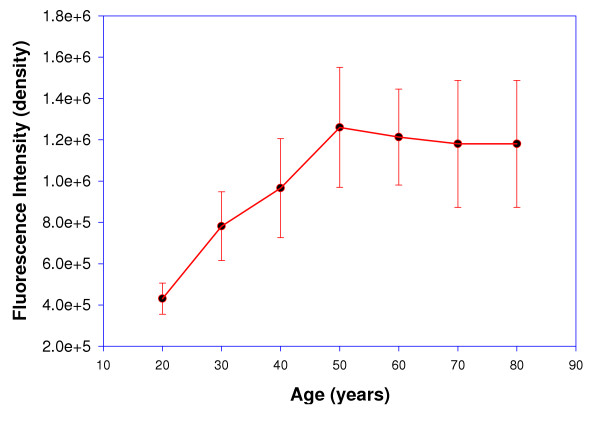
Relationship between age (20–80 years) and fluorescence intensity at λ_Ex312 nm _of intact human lenses, using digital based image analysis. (*y *-axis values in exponential; Values, mean ± S.E).

The Digital image analysis of the intact lens (20 and 60 years) is depicted in figure 2. A significant increase in the net fluorescence intensity was observed in the 60-year-old lens as compared to 20-year-old lens, which was used as a control. Figure 3, 4 depicts the net fluorescence intensity recorded in the intact lenses at λ_Ex312 nm _and λ_Ex365 nm _from the different age groups. The fluorescence intensity (density) in the intact lenses shows an increasing trend up to the age of 50 years (*r*^2 ^= 0.9 at λ_Ex312 nm _and *r*^2 ^= 0.94 at λ_Ex365 nm; _Confidence interval 95%). There was a statistically significant (p < 0.001) increase in fluorescence intensity (density) at λ_Ex312 nm _as compared to λ_Ex365 nm _for all age groups (figure 5). The quantitative estimation of AGE-like fluorophores is shown in Figure 6a, b, c and 6d for the processed lens samples and Figure 7a & b for *in vitro *synthesized AGE-like fluorophores. Distribution of AGE- like fluorophores show a similar trend in processed lens samples as well as *in vitro *synthesized AGE-like fluorophores (figure 6 &7). There was a 2.5 fold increase in fluorescence intensity at 40 years as compare to 20-year-old lenses, with an increasing trend up to 60 years in total lens homogenate (figure 6a &6b). Similar changes were also observed in soluble/insoluble protein fractions of lenses. A threefold difference in fluorescence intensity was observed at the age of 60 years as compare to 20 year old human lenses (figure 6c &6d).

**Figure 4:**
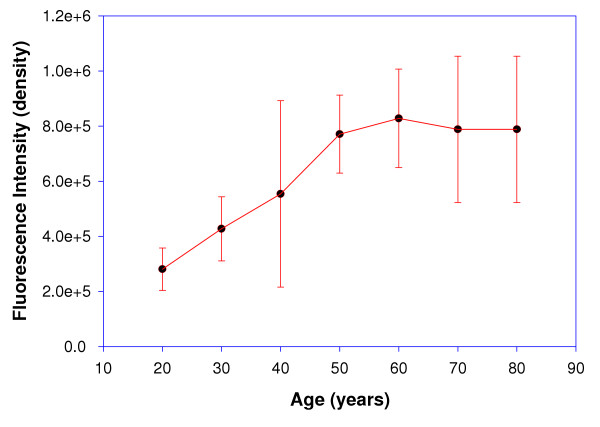
Relationship between age (20–80 years) and fluorescence intensity (λ_Ex365 nm_) of intact human lenses, using digital based image analysis. (*y -axis *values in exponential; Values, mean ± S.E).

**Figure 5:**
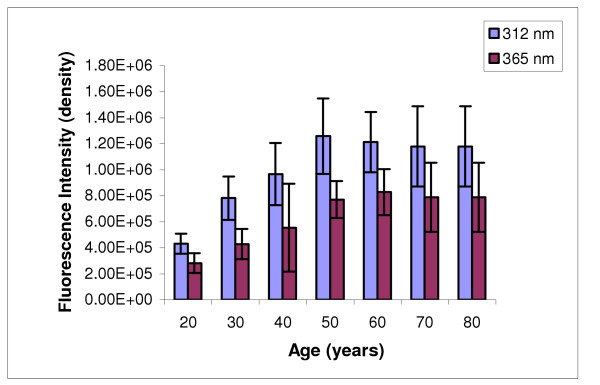
Comparison of AGE-like fluorophores (at λ_Ex312 nm _and λ_Ex365 nm_) as a function of age in intact human lenses. Increase in fluorescence intensity *(p < 0.001; Confidence interval 95%) *at λ_Ex312 nm _as compared to λ_Ex365 nm_. (*y*-axis values in exponential; Values, mean ± S.E).

**Figure 6:**
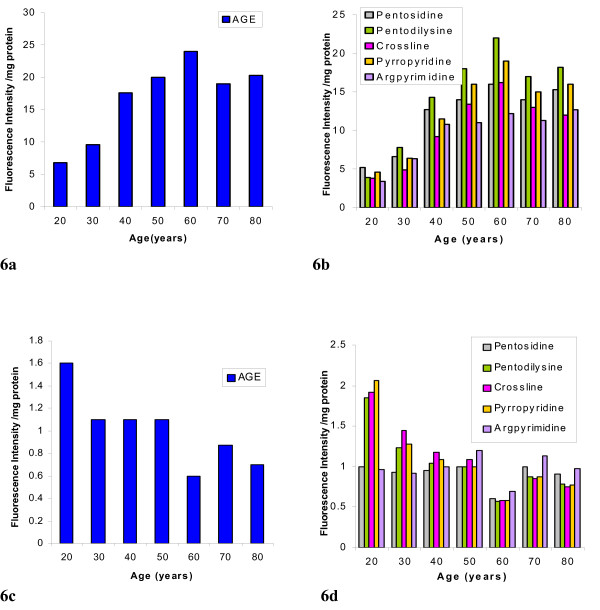
Determination of AGE-like fluorophores in processed lens (total, soluble, & and insoluble) fractions from different age group (20–80 years). Values are mean of five determinations and their CV was < 8%. **Fig. 6a **shows AGE-like flurophores in the total lens homogenate. **Fig. 6b **depicts the profile of various AGEs in the total lens homogenate. **Fig. 6c **illustrates the AGE-like fluorophores as a ratio of soluble to insoluble fraction of lens homogenate. Fig. **6d **represents the profile of various AGEs as a ratio of soluble to insoluble fraction of the lens homogenate.

**Figure 7:**
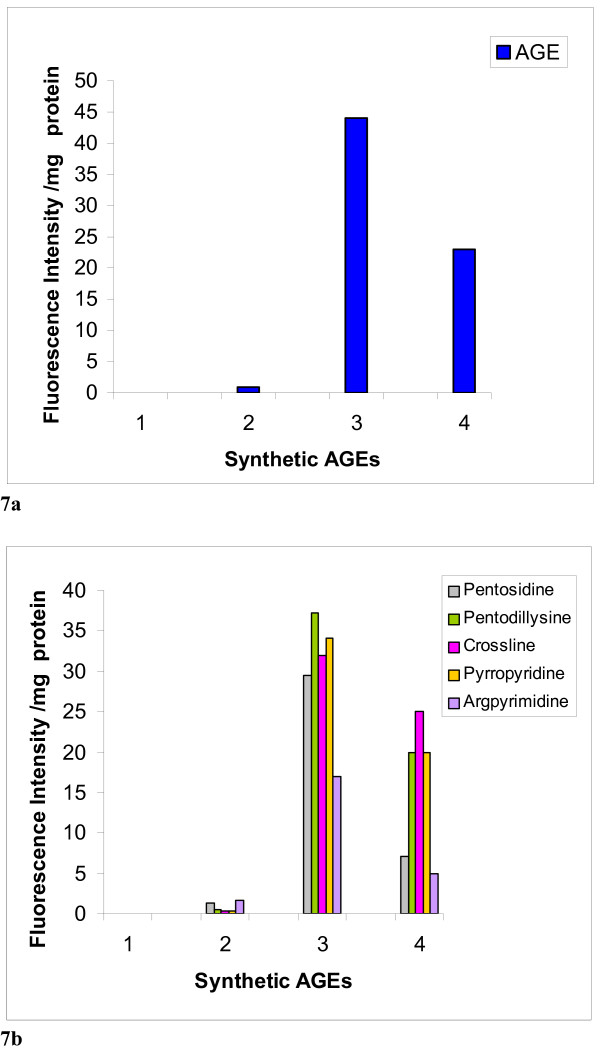
Determination of AGE-like fluorophores and various AGEs in the synthetic glycated protein (BSA) & in amino acids mixture. **Fig. 7a **depicts AGE-like fluorophores and **Fig. 7b **illustrates profile of various synthetic AGEs. (**1**) Control blank of amino acid mixture, (**2**) BSA blank, (**3) **BSA-glycated, and (**4**) Amino acid mixture-glycated.

Chemically, BSA-AGE and amino acid based AGE was successfully synthesized and tested by fluorimetric analysis. The degree of BSA-glycation was monitored by TNBS assay and was observed to be 86% at the end of 10 weeks of incubation. The BSA-AGE and amino acid mixture-AGE showed typical fluorescence similar to that of lens AGEs, while the BSA and amino acid mixture control blank, showed no fluorescence (figure 7a &7b).

## Discussion

Earlier workers have reported the presence of light absorbing chromophores formed during the course of cataract development [1]. Young lenses show higher absorbance at 280 nm due to the presence of aromatic amino acids like tryptophan and tyrosine constituents of crystallins [2]. Apart from this absorbance, the sensitive method of scanning intact lenses, based on 1024 diode array detector system, used by us indicates that the absorbance due to other suspected aromatic amino acids like histidine (240 nm), phenylalanine (254 nm) and sulphur containing amino acid like cysteine (235 nm) [34] may also contribute to the absorbance as observed in the present investigation (figure 1). This process in turn ultimately affects the shifting of wavelength from λ_378 nm _to _λ470 nm _in lenses from the age of 45 years onwards, may be due to the formation of AGE-like fluorophores. The direct relationship between the amount of AGE-like fluorophores and increased yellowing of lens was reported earlier by Das *et al*., [26] using synchronous fluorescence (SF) method between wavelengths 340 – 480 nm. The spectral data obtained in the present investigation suggests that there was an altered absorptivity in the region of λ_210_-λ_470 nm _during the course of aging. However, this interpretation is limited due to the use of solid sample (intact lens) for analysis, wherein the absorbency may reach a point of saturation at lower wavelengths (blue-end region of the spectra). Figure 1, depicts a spectral shift from blue-end cut-off towards the red end region of the spectrum, with age. Further, the ratio of the absorbance observed at λ_511–520 nm _*versus *the maximum absorbance recorded at blue-end cut-off indicates a progressive increase, with age (Table 2). As in younger lenses the absorption was minimum at 511–520 nm and because no significant change with age at longer wavelength, we chose to use the red-end tail of the UV-absorption peak of the intact lens for calculating the ratio of absorptivity at λ_(*511–520 nm*_) versus the maximum absorbance recorded at blue-end cut-off (210–470 nm), during the course of ageing. Possibly, these changes indirectly reflect the AGE-like complex formation from the age of 45 year onwards (Table 2). Lerman and Borkman [4] observed two-age related fluorescent compounds, which develop in the lens nucleus. The first showed an excitation at 340–360 nm with emission at 420–440 nm. The second, which appeared to be a secondary product of the former being detectable only after the first decade of life, absorbing light at 415 nm–435 nm with emission at 500–520 nm. It remained relatively at low level until the fourth or fifth decade. The presence of a second type of fluorophore as reported by Lerman & Borkman [4], which remained at low level (absorbance value < 1.0) until the third decade (age 30 years) of life. We observed no significant spectral changes below 20-year-old lenses, thus the fluorescence studies were carried out on lenses from 20 years onwards.

It is contentious as to whether fluorescence studies are true representative of absorption by specific fluorescent compounds present, particularly in the aging lenses. Thus, a study of intact lens, preserving all its condensed phase features, would be of interest. To quantify this age related changes, the fluorescence studies were carried out at two wavelengths - λ_Ex312 nm _and λ_Ex365 nm _(figure 2,3,4). The results obtained clearly demonstrate that as the human lens ages, there were significant changes in the non-tryptophan (or "blue) fluorescence [22] because both selected wave length are for blue fluorophore compounds like argpyrimidine[18] and pentodilysine[35]. There are undoubtedly a number of post-translational modifications that occur in lenses with age. The change of AGE- like fluorophore in lenses were found to increase until the age of 50 years, there after, fluorescence begins to drop. A probable explanation for this observation is that a method of external fluorescence measurement on the intact lens cannot follow the increasing concentration of fluorophores because it is affected by increasing self absorption (figure 3 & 4) at λ_Ex312 nm _and λ_Ex365 nm_, respectively with age. These observations indirectly decipher the characteristic trough observed at λ_467–556 nm _(figure 1) in aging lenses. Previous studies have shown that lens auto-fluorescence increases quantitatively with age [21,22] and in diabetic condition [22]. The remarkable similarities between diabetic and the non-diabetic lenses suggest that the mechanisms of fluorophore formation are alike in diabetic and non-diabetic condition. Increased glycation of lens crystallins in senile and diabetic cataractous lenses have been investigated by immunochemical and fluorescence studies [19,26]. These, findings indicate fluorescent AGEs species including pentosidine [22], pyralline [24], crossaline [23], vesperlysine, and argpyrimidine [24] in lens. Undoubtedly, there are a number of AGE-like fluorophore that are formed in human lenses during the course of aging. The rate of change of λ_Ex312 nm _/λ_Ex365 nm _may be considered to be a qualitative estimate of argpyrimidine (λ_Ex320 nm_) and pentodilysine (λ_Ex366 nm_), which may reflect their relative level during ageing. The level of argpyrimidine (λ_Ex320 nm_) was found to be significantly higher than any other fluorophore (Figure 5). This inference has been further supported by the observations of Kessel *et al*., [22], $wherein they reported higher concentration of argpyrimidine in cataractous lenses. It is pertinent to note that the distribution of these fluorophores in processed lens samples, did not show any distinct pattern at their respective excitation (Figure 6b &6d). This suggests that one of the dominating fluorophore or very closely related groups of fluorophores give rise to a typical pattern (Figure 6a &6c) of the fluorescence spectra in the processed lens sample. Kessel *et al*., [22] observed a similar phenomenon. The changes in these fluorophores possibly commences from 30 years of age and peaks at 60 years, when total lens proteins are take into account (figure 6a); while the trend is reversed when the ratio of soluble to insoluble lens protein fraction is considered (figure 6c).

AGE-like fluorophore concentration is found to increase in both total and insoluble fraction in the lens during the period of 40 – 60 years. (Figure 6a &6c). This finding is similar to the one reported earlier by Das *et al*., [26], using synchronous fluorescence (SF) and immunochemical methods. These observations suggest that AGE-like fluorophores may contribute to protein insolubilizaton, which ultimately leads to the coloration, opacity and catractogenesis along with the age.

Our present fluorescent data of human lens samples resemble those of chemically synthesized AGEs, derived from incubation of BSA and amino acids mixture with sugars (figure 7), may suggest that such fluorophores contribute to the fluorescence of human lens. In addition to the spectroscopic observations, biochemical basis is put forth to explain the accumulation of AGE-like fluorophore in processed lens samples, which significantly contribute to the absorption of blue light and thereby appearing yellow in aging lenses.

## Conclusion

The present experimental study clearly indicates that changes in AGE-like fluorophore in lens increases along the age and with major changes commencing from the age of 40–50 years onwards. This is a significant observation with respect to Indian human lenses as it may lead to early onset of senile cataract.

## Competing interests

The author(s) declare that they have no competing interests.

## Authors' contributions

Mala Ranjan, carried out the experimental as well as data analysis under the supervision of Dr. B. Sashidhar Rao. Both authors read and approved the final manuscript

## Pre-publication history

The pre-publication history for this paper can be accessed here:


